# The Canine Search and Adoption Decision Process: A Conceptual Framework for Companion Pet Shelter Adoption

**DOI:** 10.3390/ani16081255

**Published:** 2026-04-19

**Authors:** Lawrence Minnis, Doris Bitler Davis

**Affiliations:** Psychology Department, George Mason University, Fairfax, VA 22030, USA; dbitler@gmu.edu

**Keywords:** canine, dog, adoption, animal welfare, attachment bond, consumer behavior, decision-making, neuroeconomics

## Abstract

Understanding how individuals decide to adopt shelter dogs remains a significant challenge within animal welfare research, as existing studies identify correlates of adoption outcomes without explaining the underlying decision process. This hypothesis introduces a conceptual framework that synthesizes empirical findings from dog adoption studies with interdisciplinary theories to explain how adoption decisions emerge. Using a signal-to-noise perspective, the framework conceptualizes early bond formation between a potential adopter and a dog as a valuation signal that competes with uncertainty arising throughout the process. The functional model describes the adoption process as a lifecycle involving search, visitation, interaction, and decision phases, during which potential adopters seek information, evaluate available dogs, and form perceptions of compatibility. Interdisciplinary decision models, including Prospect Theory and the Diffusion Decision Model, are integrated to explain how information is framed, evaluated, and accumulated until a decision is reached. Empirical findings from human–dog interaction research are used to support the hypothesis that potential adopters evaluate companionship potential based on early bond formation associated with human–dog interactions. The framework offers a broad perspective on how adoption decisions may occur and establishes a theoretical foundation to guide future hypothesis development, measurement, and experimental research in companion animal adoption.

## Simple Summary

Animal welfare researchers have long needed a comprehensive theory or model that explains how people search for and decide to adopt shelter dogs. Current research identifies many factors that influence adoption, such as a dog’s age and appearance, but lacks an organizing framework that explains how these factors may impact the search and decision process. This article addresses this gap by proposing a new conceptual framework based on competing forces: factors that build emotional connections with dogs versus factors that create doubts or uncertainty about adoption. The framework integrates insights from attachment research, consumer behavior, and cognitive neuroscience (the study of neural processes) to map the complete journey from initial online searches through shelter visits to final adoption decisions. This provides researchers with a structured foundation for developing hypotheses and designing studies, while helping practitioners identify specific intervention targets that could improve adoption outcomes. The framework opens new directions for evidence-based improvements in animal welfare practice.

## 1. Introduction

Companion animal adoption research lacks a fundamental theory and model to provide a foundational view that research can reference and build upon. For nearly two decades, researchers have called for deeper examination and understanding of adopter characteristics [1,2,3], and, more recently, for modeling of the adoption decision-making process [4,5]. To develop a comprehensive model, researchers must concurrently define the parameters of the process, characterize the principal stakeholders, and identify distinct relationships and behaviors within the process [6,7]. We address all three tasks and propose this conceptual framework for integrating interdisciplinary theories with current dog adoption research, using a cognitive and behavioral neuroscience perspective. This perspective focuses on the origins of observable decision behavior to identify the cognitive mechanisms related to those behaviors. In other words, the proposed framework does not view individual behaviors in isolation but instead considers how they emerge from a broader sequence of cognitive processes that unfold throughout the adoption search and decision process.

The aim of this conceptual framework is to provide a process-focused explanation of how shelter dog adoption decisions are made. The framework integrates interdisciplinary research in order to explain both the observable behaviors involved in adoption and the cognitive processes that underlie the behaviors leading to decision [8,9]. The perspective and integration are important because observable behaviors alone cannot precisely reveal the cognitive processes that produced them, especially regarding decision-making [10].

For example, a potential adopter may report in a survey that a dog’s age was an important factor in their decision. However, that report does not clarify how age was actually used during the decision process. Age might function as a paramount factor or serve as a heuristic (mental shortcut) to infer on behavior and lifestyle compatibility that may be difficult to assess during short shelter interactions. As a result, retrospective data and self-report findings may identify age as an important factor in adoption decisions without clearly revealing the cognitive processes through which that factor influenced the actual decision.

Understanding these cognitive processes is critical in order to interpret observable behaviors reported in adoption studies. This framework positions these behaviors within the broader adoption process and provides additional context for interpreting findings from existing research. Through this perspective, it highlights the mechanisms, relationships, and decision dynamics that may appear throughout the dog adoption process and offers a baseline perspective for future research examining how adopters evaluate dogs, address uncertainty, and ultimately reach an adoption decision.

To organize these mechanisms, the framework draws on the concept of a signal-to-noise ratio (SNR) as a strictly conceptual analogy, in which the signal is the detectable evidence and noise is the factor that corrupts the evidence and increases error [8,9]. This frames our understanding of how adopters seek and interpret information within an uncertainty-laden decision process and progress toward a decision when a dog’s perceived companionship and compatibility, assessed via early bond formation, sufficiently outweigh the noise of perceived uncertainties. Here, we propose that the **signal** relates to latent internal valuation processes that emerge as potential adopters begin to form an early affective bond with an available dog based on factors such as social behavior and interaction quality. Evidence supporting this interpretation derives from research demonstrating that human–dog gaze and brief affiliative interactions can increase oxytocin (OT), a neuropeptide strongly associated with social bonding, in both humans and dogs [11,12,13,14,15,16,17]. These findings indicate that even short interactions significantly correlate with bonding-related processes between dogs and humans [18]. This interpretation diverges from the common conception of social bonds or attachment, which is developed and measured over time.

We therefore posit that early bond formation functions as a key valuation signal within the adoption decision process, emerging as potential adopters register available cues to assess the dog’s potential companionship and compatibility and attenuate the effects of uncertainty while considering which dog to select and whether to adopt that specific dog [3,19,20,21]. There is also a positive relationship between the signal and the decision to adopt. We must acknowledge that the OT findings demonstrate physiological correlations with human–dog interactions and do not directly implicate the decision valuation process. Our hypothesis infers that the underlying physiological response is associated with a valuation process that potential adopters use to evaluate an available dog as the best compatible companion relative to available alternatives.

The **noise** represents uncertainty and ambiguity that arise from factors such as incomplete information, environmental factors, and adopter-specific concerns about compatibility and future ownership outcomes. It negatively relates to the likelihood of the decision to adopt, so as uncertainty increases, the likelihood of the potential adopter reaching the decision to adopt decreases. Humans demonstrate high aversion to uncertainty and seek to reduce it [22,23] or avoid it when possible [24,25,26]. Under such conditions, the decision to adopt may emerge as adopters register available cues [27,28,29,30], form early affective bonds [12,16,31,32,33], and gradually reduce uncertainty until they reach a point where they are confident committing to an adoption, which mirrors the decision threshold within evidence accumulation models, like Diffusion Decision Model (DDM) [34,35].

This SNR perspective provides a conceptual lens for interpreting many of the findings reported in dog adoption research. Similar conceptual approaches have been used in other decision frameworks to understand individual behavior, such as DDM [34,35], which will be briefly discussed later.

Although existing studies have identified numerous correlations between variables, like morphological traits or perceived behavior, with adoption outcomes, less attention has been given to defining the variables’ functions within the broader adoption decision process. In the present framework, dog characteristics, such as morphology or behavior, are conceptualized as signal-generating inputs that influence the intensity of this bond formation process [20,21,36,37]. This interpretation is further supported by evidence from human–dog interaction studies that have showed increased OT levels for both humans and dogs following short and long social interactions periods [12,16].

To set the present framework within the existing research landscape, the following section briefly synthesizes key empirical findings from dog adoption studies. This synthesis highlights patterns in the current literature while also identifying gaps that the proposed framework is intended to address.

### 1.1. Knowledge Gaps Within Dog Adoption Research

This section briefly synthesizes key empirical findings from dog adoption research to demonstrate patterns in the research and highlight the mechanisms that this framework is designed to explain. Recent reviews [1,38] provide comprehensive summaries of adoption decision research, highlighting the correlations between morphological features, such as age or size, and behavior with adoption decisions, respectively. Both reviews state that morphological features consistently predict dogs’ length of stay and likelihood of adoption; however, there is marginal insight on their direct effects on decision behaviors and more examination is needed.

Select studies have shown some direct effects of behavior on in-kennel selection [20,37,39] and adoption decisions [19,21,40]. But there are limited insights into the conceptual nature of the decision process. Protopopova and Wynne [21] suggested a unique conception of the decision process as two or more possible levels in which decisions may occur. Table A1 (Appendix A) provides examples of journal articles published on dog adoption decisions since 2020 [41,42,43,44,45,46,47,48,49,50,51,52,53,54,55,56,57], which yielded findings that generally align with prior research. Recent research has moderately shifted to focus less on discrete, defined decision-making behaviors and more on pre-acquisition behaviors and post-acquisition outcomes. The shift in research trends likely relates to the conceptual model knowledge gap. Marginal insights into the process and mechanisms of adoption decisions have become barriers to the research questions and hypothesis development needed to guide future research.

**Figure 1:**
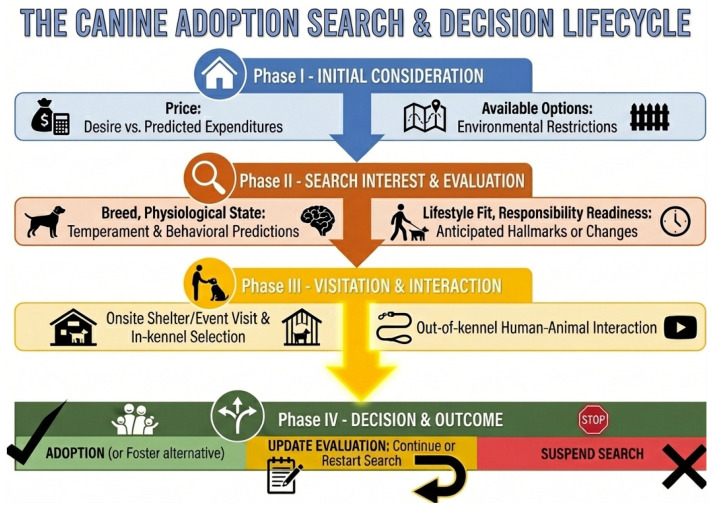
Each phase in this figure is characterized by the assessment of relevant variables specific to the adopter’s bonding mechanisms and uncertainty reduction. Additional variables may also be considered during the phase. The duration of these sequences remains idiosyncratic, dictated by the unique psychological and environmental context of the individual.

The decision process has been largely researched in relation to a singular decision point that occurs at the end of the process. Our conceptual framework aligns with Protopopova and Wynne’s [21] suggestion of a multi-level process and provides detailed insight into the factors and decision behaviors that are unique to each decision point, or level. Before progressing further, it is necessary to distinguish between the phrases: “*adoption decision*” and “*decision to adopt*”. Although both phrases are commonly used interchangeably, they reference different concepts in the present framework. The “*adoption decision*” references the final decision point of the process, which may result in one of multiple potential outcomes (Figure 1). The “*decision to adopt*” references a single, specific outcome within the final decision point, in which a dog is adopted.

### 1.2. The Need for Interdisciplinary Approach

Adopting an interdisciplinary lens prevents the duplication of existing theoretical work outside of pet adoption research. Leveraging established insights from external research fields that have previously explored the mechanics of decisions is critical and allows for a more sophisticated, efficient expansion of our current knowledge with regard to pet adoption decisions. To date, Siettou et al. [58], Vink et al. [4], and Rishi et al. [43] have provided the only models that explore decision-making processes by integrating the research literature on consumer demand theory, social cognition theory, or appraisal theory, respectively. The approaches, however, have limited scope with regard to the behavioral dynamics of the adoption decision-making process.

Neither of the interdisciplinary models is sufficiently structured to address consumer behaviors, the effects of shelter environments, or option availability, which have all been empirically shown to correlate with the final adoption decision [5,19,21,59]. Likewise, none of the models provide conceptual frameworks that explain the dynamics of the adoption search and decision process, meaning “how”, “when”, or “why” the variables affect the process or its stakeholders. Although the interdisciplinary integrations offer novel perspectives to the research field, they offer limited explanatory power to address existing knowledge gaps.

These limitations highlight the need for a conceptual framework that explicitly models the behaviors and dynamics of the adoption decision process. This present proposal begins to address these gaps by framing the relational, behavioral, and sequential dimensions of the canine adoption decision process.

### 1.3. Conceptual Framework for Canine Adoption Decision-Making

The proposed framework offers a process-focused, explanatory model that consists of the search, visitation, interaction, and decision-making phases of the pet adoption process (Figure 1). This section will provide a top-down overview of the complete process, the underlying behaviors and relationships, and the interdisciplinary concepts that define this framework. This overview begins with the main goals of potential adopters.

There is strong consensus within the dog adoption literature that the primary goal or purpose of acquiring a dog is companionship [2,54,60,61,62,63,64,65]. And compatibility is a critical component of the companionship goal [2,60,62,64,66,67]. This infers that potential adopters aim to balance their expectations, morphological preferences, cost constraints, and lifestyle in effort to add a companion dog to the household [38,63,64,68,69,70,71]. Additionally, potential adopters that prefer obtaining a dog through shelters and rescues have pointed to altruistic motivation or ethical responsibility as important factors in their overall considerations [38,64,68,69]. Collectively, these findings suggest that potential adopters value these overarching goals enough to warrant engaging uncertainties throughout the search and decision process. The commitment to the process launches the process lifecycle (Figure 1).

**Figure 2:**
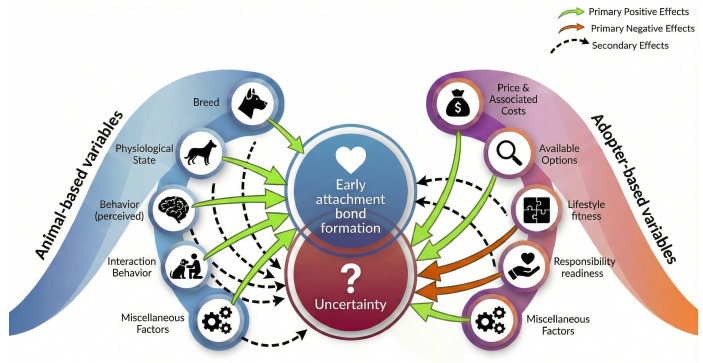
This figure illustrates that early bond formation is generated by the adopter’s response to surrounding variables within the adoption search and decision process. Additional variables may apply and are dependent upon the adopter and surrounding contexts, which are subject to change over time.

The adoption search and decision lifecycle presents a sequential view of the process that potential adopters will likely encounter after launching the process. Each phase of the lifecycle incorporates one or more signal-generating (increasing early bond formation) or noise-generating factors (increasing uncertainty) to which the potential adopters respond (Figure 2). Given the proposed signal’s positive relationship and noise’s negative relationship with the decision to adopt, potential adopters are primed to behave in manners that increase the chances of signal generation and decrease the chances of noise generation.

#### 1.3.1. Noise: Uncertainty Surrounding the Decision Process

Uncertainty is a foundational construct in decision-making research [24,72,73] and has been widely examined across domains such as consumer science [74,75]. This framework aligns with the classical theory definition of uncertainty as *a condition in which outcome alternatives are inherently ambiguous and their probabilities cannot be clearly specified, measured, or controlled* [7,24,25,72]. By this definition, the environment surrounding the adoption decision process is inherently noisy and filled with uncertainties. This may be attributable to the inherent design of the shelter system that must balance animal welfare, public safety, and pet population management responsibilities despite resource limitations [76]. Shelters and rescues can have dogs with unknown histories or background [63], misleading breed designations [77,78] that can negatively impact perception [53,79], statistically invalid behavioral assessments that may unreliably predict future in-home behavior [80,81,82], or protocols and aversive environments that may contribute to uncertainty [83,84,85,86]. But this overarching noisy environment is readily acknowledged by individuals that prioritize acquiring companion dogs through shelter/rescue adoption [38,64,69,87].

As potential adopters face uncertainty throughout the process [38,41,63,64,65,68,69,88], they demonstrate information-seeking, evaluation behaviors, and other observable behaviors that function to reduce or avoid uncertainty [24,29,89,90]. Several animal welfare studies indicate that potential adopters infer on dogs’ personality or behavior patterns using photographs, videos, breed labels, descriptions, and other information sources to develop perceptions that directly relate to a dog’s compatibility or adoption likelihood [53,56,77,79,91,92]. This suggests that during Phases I & II of the process lifecycle (Figure 1), potential adopters seek information (ex., online adoption listings, shelter websites, social media, etc.) and create perceptions that may allow them to differentiate between available dogs. That behavior aligns with the arguments within the Prospect Theory [93,94], a descriptive interdisciplinary model that explains how humans navigate the decision-making process under risk and uncertainty.

Prospect theory advances that the process comprises two phases: the *framing phase* and the *valuation phase* [73,94,95]. The framing phase is characterized by the editing of prospects, or options, based on the simplest features or representations to sort and analyze [95]. That is followed by the valuation phase, which is characterized by the value assessment of each prospect based on select features and the ultimate selection of the highest-value prospect. Under the Prospect Theory model, potential adopters frame available dogs using media resources and available information to differentiate prospects using simple features, like appearance, breed, or other features that have been empirically shown to significantly relate to perceived adoptability [53,68,77,79,91,96]. This sheds light on various functions of morphological variables within the process (Figure 2). Accordingly, potential adopter behaviors during Phases I & II function to establish the basis for the subsequent valuation phase related to Phase III.

Through the lens of Prospect Theory, potential adopter behaviors transition from functions for framing to valuation to assess available dogs and select the “highest value” dog (i.e., the most compatible companion). This implies that the mechanism for valuation should correlate with the simplest features used to sort available dogs and with the features that indicate compatibility and companionship. The mechanism must also help potential adopters address uncertainties within the search and decision process. Decision-making research suggests that individuals increasingly rely on affect for decisions under uncertainty [22,23,27,28,90], and social affect benefits that have been empirically related to human–dog interaction may provide a substantial resource to address the uncertainty of the process [11,13,16,17,36,97,98,99].

This argument finds support in the process lifecycle (Figure 1), where the system and potential adopter behaviors are centered on social observation and interaction. Phase III involves individuals touring a shelter or adoption site, observing and interacting with available dogs in the kennel, and possibly interacting privately with specific dogs outside the kennel [3,5,21]. These activities function to complete the framing phase and launch the valuation phase, described in Prospect Theory. The neuroeconomic literature has informed the framework development (Table 1) and provided insight on evaluation that may occur and that corresponds with dog adoption research [24,27,28,73,90,93,94,95,100,101,102,103,104,105,106].

Christopher Hsee has argued that all decisions are made using one or two distinct methods of evaluation: joint evaluation and separate evaluation [103,105,106]. Joint evaluation is characterized by the exposure of multiple items, or options, simultaneously and a comparative evaluation amongst all the options [105]. Separate evaluation is characterized by exposure to only one item, or option, which is evaluated in isolation. The distinction between the modes is critical because of potential joint-separate evaluation reversal, which is a phenomenon where the value or rank of features can change based on the change in evaluation mode [104,105,106,107]. This suggests that features ranked or valued within one evaluation mode may alter significantly when ranked or valued in the other mode.

Both modes align with the two stages of Phase III in the proposed model (Figure 1): in-kennel selection (joint evaluation) and out-of-kennel interaction (separate evaluation). Additionally, joint-separate evaluation reversal is implicated when reevaluating the current literature on the adoption process. For example, Protopopova and Gunter’s [1] review highlights the significance of morphology for choosing among available dogs during the in-kennel selection stage a shelter visit. However, the review subsequently states that behavior was a significantly important variable within the out-of-kennel interaction. Through the lens of Hsee’s evaluability hypothesis [103,105,106], the change in significance from morphology to behavior may result from the change from joint evaluation mode during the shelter tour to separate evaluation mode during the private out-of-kennel interaction.

Altogether, this suggests that behavior represents the simplest feature to assess compatibility and companionship. The dog adoption literature has also highlights in-kennel behaviors that relate to adoption likelihood indicators [3,37,108,109]. This infers that both morphology and in-kennel behaviors affect adoption likelihood during the joint evaluation in Phase III, by providing information to the potential adopter on compatibility, companionship, and the potential tradeoffs needed post-adoption. But only behavior has been shown to predict adoption likelihood in the separate evaluation, suggesting that it is the only factor that sufficiently predicts compatibility and companionship.

#### 1.3.2. Signal Leading to Adoption Decision

Thus far, we have discussed the effects of uncertainty within the search and decision process and the functions that potential adopter behaviors may serve to address uncertainty in pursuit of a larger adoption goal, compatible companionship. We have also posited the role that early bond formation plays in the decision process. This section provides additional support for classifying early bond formation as the valuation signal in this process, leading to an adoption decision.

First, early bond formation, as the valuation signal, may allow potential adopters to confidently predict compatibility and the tradeoffs that may be needed if the selected dog is adopted [38,63,64,66,69,70], as well as feel a sense of companionship that can develop over time [66,110,111]. In the present framework, the latent cognitive process underlying bond formation is manifested as the effect of human–dog interaction on OT and observable social behaviors, like talking to a dog and extended human–dog gazing [11,13,14,15,16]. This is an important pathway that is empirically valid, measurable, and modifiable [11,12,15,32,33,112]. We posit that the OT activity and observable social behaviors directly relate to long-term human–dog bond or attachment and relationship satisfaction that are targeted by existing attachment measures [113,114,115,116,117] through the shared underlying latent process.

As previously described, potential adopters seek information while progressing through the uncertainty of the decision process in order to detect early bond formation, which helps them evaluate available dogs and mitigate the effects of uncertainty. However, Prospect Theory and the evaluability hypothesis do not sufficiently resolve the mechanisms that allow the potential adopter to reach a decision within Phase IV. Another interdisciplinary decision model, the Diffusion Decision Model (DDM), provides conceptual support in this matter.

The Diffusion Decision Model, a type of evidence accumulation model, defines decision-making as a process in which an individual iteratively accumulates evidence in favor of an option or alternative until enough evidence is gathered to reach a latent threshold, after which a decision is made [34,35,118]. This description comparably aligns with the structure of the present framework. Likewise, DDM assumes that the process is noisy, which may impact the decision performance and outcome [34,35,118]. From this conceptual perspective, a potential adopter iteratively seeks and acquires information on available options until a latent threshold is reached regarding early bond formation, prompting a decision and corresponding action.

We hypothesize that, as sufficient bond formation occurs, the underlying bond threshold will be achieved and lead potential adopters to reach the final decision point of the process (Phase IV). In order for sufficient bond formation to occur, we propose that potential adopters accumulate information through iterative interactions with an available dog. We also propose that longer interactions would result in increased bond formation, based on the hypothesized valuation function related to increased OT. For potential adopters, the bond formation would be valued enough for them to expend time, energy, and money to progress through the process. Adoption research provides some support for these hypotheses and expectations.

Wells & Hepper [3] observed several behavioral distinctions for adopters and for lone visitors. First, adopters spent significantly more time in front of and interacting with the dog they adopted compared to other dogs they visited, which fits the stated expectations. Interestingly, lone visitors, on average, visited significantly more dogs, spend longer in front of kennels, and prompted interaction with a dog more frequently than visitor pairs or groups. This suggests that lone visitors may have valued the early bond development noticeably more than visitor pairs or groups.

Likewise, Protopopova & Wynne [21] observed out-of-kennel interactions and noted statistically greater likelihood of adoption for dogs that actively engaged in play and spent more time in close spatial proximity to the potential adopter. Conversely, dogs that ignored play cues had significantly less likelihood of adoption. Additionally, the researchers noted that adoptions were more likely in smaller areas that larger areas and argued that smaller settings could be more conducive to social interactions. Drawing on those findings, a structured meet-and-greet intervention was tested to explore if the intervention could increase adoption rates [19]. The results showed the intervention successfully increased human–dog social plan and time in proximity and decreased less independent dog behavior, with dogs in the intervention group experiencing higher adoption rates. These findings provide preliminary support for the stated expectations and assumptions of this framework. Similar research has indicated prosocial in-kennel and out-of-kennel dog behaviors, like approaching or seeking contact, related significantly with adoption indicators [5,59,108].

Ultimately, the framework articulates relationships and assumptions that find support within the existing dog adoption literature and provides an interpretation of some adoption data that can address existing knowledge gaps by conveying the relational dimensions (“why?”) of the decision process, defining the relationships between variables, and providing sequential and behavioral dimensions (“when?” and “how?”) using insights from the interdisciplinary decision models. The next section suggests how the framework broadens the frontier for future dog adoption research.

## 2. Future Implications

The present framework identifies two central, latent internal variables (early bond formation & uncertainty) that may predict a potential adopter’s decision likelihood. This is significant, as both latent variables have preexisting, validated measures for manifest variable that can inform measure development in this research field [113,114,115,119,120,121] or be modified to use at different points within the search and decision lifecycle. Moreover, both latent variables relate to both adoption and relinquishment decisions. This means that individuals can potentially be measured for both bond and uncertainty related to an adoptable dog or existing companion dog across multiple points along the companion pet adoption lifecycle to predict several actions or outcomes, such as adoption, relinquishment, training, or euthanasia. Furthermore, the framework could be adapted to fit processes related to other companion animal species.

The sequential breakdown of the decision process may allow researchers to control the reference points, evaluation mode, and other systematic differences within the scope of the process provided within the proposed framework. We suggest that future research target specific points, phases, and/or behaviors to investigate research questions and test the framework and arguments behind this proposal. It is expressly important for future research to clearly define which decision point or outcome behavior is the focus of the research.

For example, if a survey asks an adopter why they choose their adopted dog, it may prime the adopter to reference the visitation & in-kennel selection (Phase 3) point of the process (Figure 1). However, if the survey asks the same adopter how or why they decided to adopt the dog, it may prime the adopter to reference the out-of-kennel interaction within Phase 3. Given the distinct evaluations modes and potential for joint/separate evaluation reversal, it may be best practice for future research to explicitly specify the in-kennel selection or out-of-kennel context within a survey or other data collection instrument.

The framework also provides some shelters and rescues with a central point of reference for decisions regarding dog adoption. Because the framework is centered on early bond formation as the valuation signal for an adoption decision, organization strategies and decisions can be discussed with specific regard to the potential impact on bond formation or on uncertainty. For example, a shelter may consider the impact of fully enclosed, solid wall kennel designs, compared to modular mesh and chain link designs, on human–dog in-kennel interaction and subsequent effects on early bond development as it may relate to adoption rates. This may provide further insight into operational tradeoffs such as disease management, liability risks and costs, adoption rates, and fundraising impacts.

Organizations can also assess the adoption of protocols and interventions comparably by referring to the impacts on early bond formation or uncertainty. For instance, a multisite feasibility study to test the implementation and effects of structure meet-and-greet interactions encountered opposition and apathy for the new methods that created unexpected barriers for a design that demonstrated early positive effect on both early bond formation and related adoption rates [40]. This framework may provide a simple but functional purpose for future efforts: “to increase the early bond formation between dogs and potential adopters under the best conditions for the decision threshold to be reached”.

This framework provides a substantial guide for future research and practical applications, but there are limitations. First, the framework is untested and needs substantial direct examination to challenge, update, or validate the model. To the best of our knowledge, no measure has been developed to validly define a range or threshold value that significantly correlates or predicts a decision to adopt. Therefore, substantial measure development and testing is needed in future research.

## 3. Conclusions

To the best of our knowledge, no prior conceptual model or theory has been published that comprehensively addresses the relational (why?), behavioral (how?), and sequential (when?) dimensions of the dog adoption decision process. The present framework addresses these dimensions while drawing a linear relationship between significant predictor variables in the current literature, the early development of social bonds, the effects of uncertainty, and the outcomes for adoption decisions.

The integration of the neuroeconomic literature provides further insight into the mechanisms and behaviors that underlie the findings within current research. Future research should no longer reference dog adoption decisions as a simplistic, aggregate process when evidence from dog adoptions studies and neuroeconomics suggests the presence of disparate behaviors, decision points, and phases over time. This framework provides a conceptual reference and offers the next steps forward, although there are initial limitations. There is substantial promise for innovation and research to occur moving forward, and there is abundant opportunity to further inform the applied humane and animal welfare community with an established foundation for understanding the operational mechanics within canine adoption search and decision processes.

## Figures and Tables

**Table 1:** Interdisciplinary Theories Informing Adoption-Related Decision-Making Under Conditions of Uncertainty.

Theory/Framework	Key Authors (Year)	Core Decision Mechanism	Primary Contribution to Decision Science	Contribution to Proposed Framework
Prospect Theory	Kahneman & Tversky (1979) [94]	Two-stage process: framing (editing) followed by valuation	Demonstrated that decisions deviate systematically from normative rational models due to framing effects, loss aversion, and reference dependence	Provides a foundational model for understanding how adopters evaluate dogs based on selectively attended cues under uncertainty
Framing Effects	Tversky & Kahneman (1981, 1992) [73,95]	Selective editing of options based on salient features	Showed that equivalent outcomes produce different choices when framed differently	Explains why certain dog attributes (e.g., behavior, age descriptors) disproportionately affects decisions
Joint-Separate Evaluation	Hsee (1996, 2000) [105,106]	Attribute evaluation differs depending on comparative context	Identified systematic preference reversals depending on whether options are evaluated jointly or in isolation	Relevant to shelter contexts where visitors may see dogs in isolation or comparatively
Evaluability Hypothesis	Hsee et al. (1999) [104]; Hsee & Zhang (2010) [103]	Attributes vary in influence based on ease of evaluation	Demonstrated that hard-to-evaluate attributes gain weight in joint evaluation	Helps explain why observable cues (size, activity, appearance) dominate adoption decisions over features
Affective Decision-Making	Rad & Pham (2017) [28]	Emotional responses guide preference formation	Integrated affect as a driver of choice beyond cognitive evaluation	Aligns with emotionally charged shelter environments where affect may amplify or mitigate uncertainty
Consumer Bias & Choice Overload	Scheibehenne et al. (2010) [100]; Bar-Anan et al. (2009) [27]	Cognitive constraints under multiple options	Challenged linear assumptions about choice overload and rational comparison	Applicable to shelter visitation contexts where multiple dogs may increase reliance on heuristics
Prospect Theory Extensions	Barberis (2013) [93]; White et al. (2020) [101]; Suo et al. (2020) [102]	Context-dependent valuation and reference points	Extended prospect theory to dynamic and applied decision domains	Supports application of prospect theory principles to real-world adoption decisions
Uncertainty vs. Risk Distinction	Knight (1921) [24]	Differentiation between measurable risk and unmeasurable uncertainty	Established the foundational distinction that uncertainty cannot be probabilistically quantified	Directly applies to pet adoptions where adopters lack reliable probabilities about future behavior, health, and compatibility
Adaptive Information Sampling Under Uncertainty	Trimmer et al. (2011) [90]	Strategic information seeking to reduce uncertainty	Demonstrated that organisms adaptively adjust information acquisition under uncertain environments	Provides a theoretical basis for adopters’ cue-focused information seeking (e.g., behavior, activity) as an uncertainty-reduction strategy

Note. Variable descriptions were not provided to participants during site surveys, so possible variance in subject interpretation is present within the study. The study’s intent was to examine the top variables that visitors reportedly considered for their respective decisions, so between-subject variance in variable definition considered within the scope of study. Costs variable was included primarily due to frequent consideration within canine relinquishment research; variable seldomly included within animal shelter adoption research.

## Data Availability

No new data were created or analyzed in this article. Data sharing is not applicable to this article.

## Abbreviations

The following abbreviations are used in this manuscript:

WTP	Willingness-to-Pay
SEM	Structural Equation Modeling
SNR	Signal-to-Noise Ratio

## Appendix A

**Table A1:** This table is a list of recent empirical studies on dog adoption decision-making conducted between 2020 and 2025. Collectively, these studies extend some earlier research findings [2,8] by documenting consistent reliance on observable morphologic cues and affective influences in dog adoption decisions across contexts. However, most studies rely on cross-sectional between-subject or retrospective designs, limiting inference about within-person cue weighting or effects of uncertainty.

Author(s) & Year	Title	Core Topic	Primary Focus
Archer et al. (2025) [53]	The role of breed and personality descriptions in influencing perceptions of shelter dog adoptability	Adoption preferences; descriptive framing	Individual decision making
Cain et al. (2020) [47]	Phenotypic characteristics associated with shelter dog adoption in the United States	Predictors of adoption	Group-level predictors
Dinwoodie et al. (2022) [54]	Selection factors influencing eventual owner satisfaction about pet dog adoption	Adoption decisions; post-adoption outcomes	Individual decision making
Hoffman et al. (2021) [50]	Characterizing pet acquisition and retention during the COVID-19 pandemic	Contextual pre-adoption influences on acquisition	Group-level differences
Kelling et al. (2024) [52]	Exploring best practices in constructing dog adoption advertisements	Information presentation; marketing	Practical Applications
Kremer & Neal (2024) [51]	Where do they come from and where do they go?—Socioeconomic patterns in dog acquisition and rehoming	Socioeconomic predictors of pre-adoption behavior	Group-level differences
Lamb et al. (2021) [56]	Artificial photo backgrounds and shelter dog profile clicking and the perception of sociability	Visual cues; perceptual bias during pre-adoption period	Individual search behavior
Mead et al. (2023) [41]	“Do your homework as your heart takes over when you go looking”—Factors associated with pre-acquisition information-seeking among UK dog owners	Information seeking; uncertainty reduction during pre-adoption period	Individual search behavior
Mead et al. (2024) [55]	UK dog owners’ pre-acquisition information- and advice-seeking—A mixed methods study	Information seeking; uncertainty reduction during pre-adoption	Individual search behavior
Mesarcova et al. (2021) [46]	Good-looking vs. obedient, which would you rather take home?—Appearance vs. obedience in shelter dog adoption (Slovakia)	Comparative cue weighting for predictors of adoption	Individual decision making
Minnis et al. (2024) [42]	Decision factors considered during shelter visitation	Pre-adoption decision variables	Individual decision making
Reese (2021) [45]	Make me a match—Prevalence and outcomes associated with matching programs in dog adoptions	Decision support systems during pre-adoption period	Practical Applications
Rishi et al. (2024) [43]	Consumer willingness to buy and pay for dog-human companionship—A combination of SEM and NCA approaches	Valuation; consumer decision models	Individual decision making
Skrzypek & Zawojska (2025) [49]	What characteristics of dogs help them stay shorter in shelters?—Evidence from a Polish animal shelter	Predictors of adoption	Group-level predictors
Stephens-Lewis & Schenke (2025) [48]	Obedient, but cheeky’—Human expectations of canine behaviour and companionship	Expectation formation	Individual search behavior
Udvarhelyi-Tóth et al. (2024) [44]	Why do people choose a particular dog? A mixed-methods analysis of factors owners consider important when acquiring a dog, on a convenience sample of Austrian pet dog owners	Mixed-methods acquisition factors during pre-adoption period	Individual search behavior
Yim et al. (2025) [57]	Pet dog choice in Hong Kong and Mainland China—Exploring owners’ motivations, behaviours, and perceptions	Cultural differences in adoption	Group-level differences
